# Supplementary figures and images for: Calcium Pyrophosphate Dihydrate Crystals Increase the Granulocyte/Monocyte Progenitor (GMP) and Enhance Granulocyte and Monocyte Differentiation In Vivo
Source: Int J Mol Sci. 2020 Dec 29;22(1):262. doi: 10.3390/ijms22010262 (PMC7794855; doi:10.3390/ijms22010262)

Supplementary Figure S1. Onai

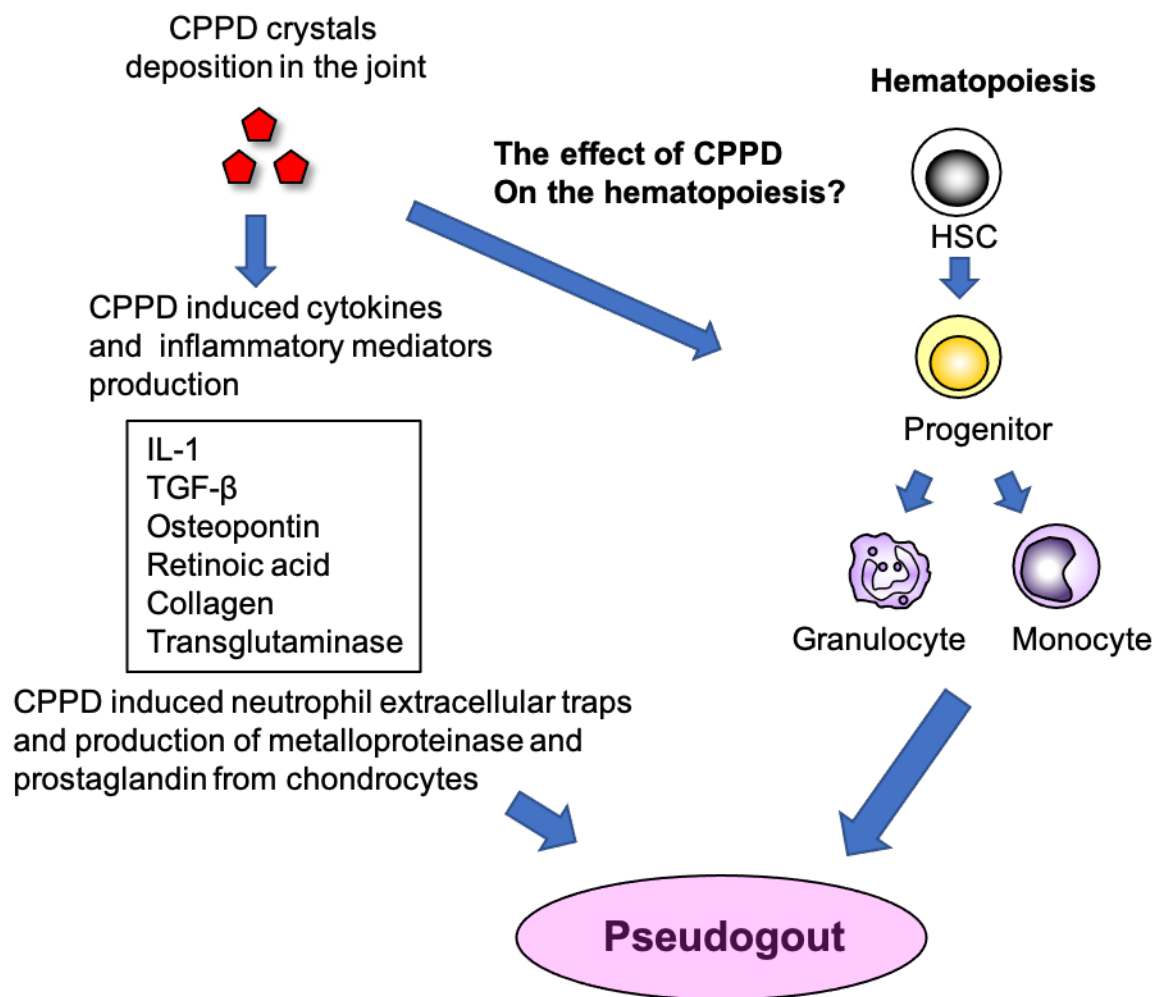

Supplement: Supplementary file 1 [file ijms-22-00262-s001.pdf]
